# Early biochemical outcomes following PSMA guided approach for bIoCHEmical relapse after prostatectomy-PSICHE trial (NCT05022914): preliminary results

**DOI:** 10.1007/s10585-023-10204-y

**Published:** 2023-04-03

**Authors:** Giulio Francolini, Michele Ganovelli, Vanessa Di Cataldo, Beatrice Detti, Saverio Caini, Mauro Loi, Gabriele Simontacchi, Isacco Desideri, Daniela Greto, Marianna Valzano, Sergio Serni, Luca Vaggelli, Viola Salvestrini, Luca Visani, Carlotta Becherini, Emanuela Olmetto, Ciro Franzese, Davide Baldaccini, Marta Scorsetti, Martina Sollini, Arturo Chiti, Icro Meattini, Richard K. Valicenti, Lorenzo Livi

**Affiliations:** 1 Radiation Oncology Unit, Azienda Ospedaliero-Universitaria Careggi, Florence, Italy; 2 Department of Biomedical, Experimental and Clinical Sciences, Serio” University of Florence, Florence, Italy; 3 Cancer Risk Factors and Lifestyle Epidemiology Unit, Institute for Cancer Research, Prevention and Clinical Network (ISPRO), Florence, Italy; 4 Unit of Urological Robotic Surgery and Renal Transplantation, University of Florence, Careggi Hospital, Florence, Italy; 5 Department of Experimental and Clinical Medicine, University of Florence, Florence, Italy; 6 Nuclear Medicine Division, Careggi University Hospital, Florence, Italy; 7 CyberKnife Center, Istituto Fiorentino di Cura e Assistenza (IFCA), Florence, Italy; 8 Department of Biomedical Sciences, Humanitas University, Via Rita Levi Montalcini 4, 20090 Pieve Emanuele, Milan, Italy; 9 Nuclear Medicine Department, IRCCS San Raffaele, Milan, Italy; 10 Department of Radiation Oncology, UC Davis, Davis, CA USA; 11 Radiation Oncology Department, AOU Careggi, Viale Morgagni 85, Florence, 50134 Italy

**Keywords:** Prostate, Radiotherapy, Bladder, Kidney

## Abstract

PSICHE (NCT05022914) is a prospective trial to test a [^68^Ga]Ga- PSMA-11 PET/CT imaging tailored strategy. All evaluable patients had biochemical relapse after surgery and underwent centralized [^68^Ga]Ga-PSMA-11 PET/CT imaging. The treatment was performed according pre-defined criteria. Observation and re-staging at further PSA progression were proposed to patients with negative PSMA and previous postoperative RT. Prostate bed SRT was proposed to all patients with a negative staging or positive imaging within prostate bed. Stereotactic body radiotherapy (SBRT) to all sites of disease was used for all patients with pelvic nodal recurrence (nodal disease < 2 cm under aortic bifurcation) or oligometastatic disease. At 3 months after treatment, 54.7% of patients had a complete biochemical response Only 2 patients experienced G2 Genitourinary toxicity. No G2 Gastrointestinal toxicity was recorded. A PSMA targeted treatment strategy led to encouraging results and was well tolerated.

**Supplementary Information:**

The online version contains supplementary material available at 10.1007/s10585-023-10204-y.

## Background

Men with biochemical relapse (BR) after radical prostatectomy (RP) are currently managed with salvage radiotherapy (SRT) [1]. However, Next Generation Imaging (NGI) has been shown to significantly impact management of treatment after BR in one randomized controlled trial [2]. Guidelines recommend to tailor treatment on the basis of Prostate Specific Membrane (PSMA) imaging [3], but there are no standard option. Although prostate bed SRT is a reasonable option for patients with negative imaging or local recurrence within prostate bed, how best to manage oligometastatic patients identified by PSMA PET imaging requires additional investigation [3]. The role of metastasis directed therapy (especially Stereotactic Body RT-SBRT) has been addressed recently in a pooled analysis from two prospective randomized trials [4], but no prospective data exploring clinical ouctomes after all forms of salvage treatment based on NGI staging is currently available. PSICHE (NCT05022914) is a prospective observational trial with the aim to explore oncological results of a [^68^Ga]Ga-PSMA-11 PET/CT tailored strategy based on a pre-defined treatment algorithm. The detection rates and their impact on treatment decisions after [^68^Ga]Ga-PSMA-11 PET/CT were previously reported [5], In the current report we present early biochemical results after NGI guided treatment administration.

## Methods

### Study design

PSICHE (NCT05022914) is an ongoing, prospective observational multicenter study at two italian centers. The inclusion criteria are prior RP with or without postoperative prostate bed radiotherapy, histological proven prostate adenocarcinoma, BR (defined as PSA > 0.2 ng/ml) but PSA < 1 ng/ml. Any patient who had a persistently elevated PSA (> 0.1 ng/ml) within 16 weeks after RP or prior SBRT to pelvic or extrapelvic disease was excluded from the trial. Postoperative androgen deprivation therapy (ADT) was discontinued at least 6 months before study enrollment. According to PSMA-RADS, a lesion was considered positive if high levels of radiotracer uptake and corresponding anatomic findings that are indicative of the presence of PCa were detected [6]. All patients were staged with centralized [^68^Ga]Ga-PSMA-11 PET/CT and treated with a pre-defined criteria based on PET/CT findings. All patients who had prior postoperative RT and had negative PSMA PET/CT imaging were managed with observation and re-staging at PSA progression. Prostate bed SRT was offered to all patients without detectable disease or positive imaging confined within the prostate bed. Stereotactic body radiotherapy (SBRT) to all sites of disease was offered to all patients with pelvic nodal recurrence (nodal disease < 2 cm under aortic bifurcation) or oligometastatic disease (≤ 3 non visceral metastatic lesions according to Italian Association of Radiotherapy and Clinical oncology (AIRO) definition [7]). Non oligometastatic disease was treated with Androgen deprivation therapy with or without other systemic treatment for hormone sensitive prostate cancer.

### Study procedures

For prostate bed SRT, The Clinical Target Volume (CTV) was defined according to European Organisation for Research and Treatment of Cancer (EORTC) guidelines [8]. A CTV to Planning Target Volume (PTV) margin of 5–8 mm was added according to clinical practice and Image guidance protocols used. Doses and fractionation schedules were according to local clinical practice, provided that at least 66 Gy in 33 fractions, or equivalent dose/fractionation regimens, were delivered. Dose intensification (e.g. 72–74 Gy in 36–37 fractions or equivalent dose/fractionation schedules) were allowed if PSMA/PET-CT findings were confined to the prostatic fossa. The target volume goal was that at least 95% of the PTV was covered by 100% of prescription isodose. Dose constraints to Organs at risk (OAR) were according to the QUANTEC-report [9]. SBRT was delivered in all patients with pelvic nodal or oligometastatic disease. The gross target volume (GTV) was any demonstrable abdominal nodal or bone metastatic disease detected by PSMA PET/CT. Morphological, topographical and metabolic informations were included in delineation of the target volumes. A GTV to PTV margin of 3–5 mm was added according to clinical practice and institutional image guidance protocols. Dose and fractionation schedules were 24–36 Gy in 3 fractions, 30–45 Gy in 5 fractions, 36 or 45 Gy in 6 fractions. Concomitant ADT was not allowed in patients undergoing SRT or SBRT. Lifelong ADTwith or without androgen receptor targeted agents (Apalutamide or Enzalutamide) was prescribed when widespread metastatic disease was detected. Complete protocol as approved by ethical committee can be found in the supplementary materials.

### Clinical outcomes

Complete Biochemical response (CBR) or partial Biochemical Response (PBR) were defined as a PSA reduction to less than 0.2 ng/ml or ≤ 50% as compared to baseline, respectively PSA decline was defined as any PSA decline from baseline as the difference between baseline and PSA three months after treatment. Biochemical progression was defined as a PSA increase above 0.2 ng/ml for patients with a PSA nadir < 0.2 ng/ml or 2 consecutive PSA increases > 25% if compared to nadir in patients with a PSA nadir > 0.2 ng/ml. Any change in PSA not meeting these criteria for response or progression was defined as stable disease. Radiological progression was defined as the occurrence of new lesion detectable with PSMA PET/CT performed in case of biochemical or clinical progression Adverse events are reported according to Common Terminology Criteria for Adverse Events v. 4.03 [10].

### Statistics

Descriptive statistic was used to report outcomes in terms of PSMA results and following treatment received, early PSA response and adverse events reported.

## Results

### PSMA results and treatment received

Enrollment began on 19/03/2021. To date, 104 patients have been enrolled, with a median baseline PSA of 0.39 ng/ml. Baseline features are summarized in Table 1. pT3a was the most represented T stage cathegory at baseline (56.7% of the whole cohort), with only 6 patients having N1 disease after surgery. A significant percentage (37.5%) of patients had GLeason 8 or higher at baseline.

**Table 1:** Principal population features

Year of Radical Prostatectomy (range)	2002–2022
**Initial PSA at Prostatectomy** **(median value, IQR)**	8.1 ng/ml (IQR 5.4–11.6)
**pT**	**T2**: 27 (26%) **T3a**: 59 (56.7%) **T3b**: 18 (17.3)
**pN**	**pN0**:71 (68.3%) **pN1**: 6 (5.7%) **pNx**:27 (26%)
**Initial Gleason Grade group**	**3 + 3**: 4 (3.8%) **3 + 4**: 28 (27%) **4 + 3**: 33 (31.7%) **4 + 4**: 24 (23%) **4 + 5**: 13 (12.5%) **5 + 4**: 2 (2%)
**Surgical Margin status**	**R0**: 50 **R1**: 54
**Time between prostatectomy and recurrence (Median, IQR)**	40 months (13-75.7)
**Age (median value, IQR)**	69 (62–72)
**PSA at recurrence (median value, IQR)**	0.39 ng/ml (IQR 0.29–0.51)
**PSMA results**	**Negative/positive within prostate bed**: 67 (64.4%)/8 (7.7%) **Pelvic nodal disease**: 23 (22.1%) **Extrapelvic metastatic disease**: 6 (5.8%)
**Number of lesions**	**0**: 75 (72.1%) **1**: 23 (22.1%) **> 1**: 6 (5.8%)
**Lesion site**	**Nodal**: 22 (75.9%) **Bone**: 6 (20.7%) **Visceral**: 1 (3.4%)

About half of the patients had positive surgical margin (R1), with a median time from surgery to biochemical recurrence of 40 months. Overall, PSMA results were negative or positive in the prostatic fossa in 67 or 8 (7.7%) patients, while pelvic nodal or extrapelvic metastatic disease were detected in 23 (22.1%) and 6 (5.76%) patients, respectively. Twenty-three patients had a single lesion detected, while 6 patients had more than 1 lesion detected. Vast majority of patients had nodal disease (n = 22), while bone and visceral metastases were detected in 6 and 1 patients, respectively Twenty two patients were observed after re-staging (due to negative PET PSMA results in patients previously treated with postoperative RT) and were excluded from the current analysis. The treatment provided was SRT, SBRT or ADT in 50 (48.1%), 29 (27.8%) and 3 (2.9%) patients, respectively. The details of the treatment are summarized in Table 2. A Sankey Diagram can be found in supplementary materials.

**Table 2:** Treatment strategy after PSMA staging and biochemical outcomes

Post PSMA treatment	Observation: 22 (21.2%) Prostate bed RT: 50 (48.1%) SBRT to nodal disease: 23 (22.1%) SBRT to Extrapelvic disease: 6 (5.7%) ADT:3 (2.9%)
**Post treatment response**	**PBR**: 33 (62.3%) **CBR**: 29 (54.7%) **PSA progression**: 5 (9.4%) **Stable disease**: 15 (28.3%)

Notes: PBR: partial biochemical response; CBR: complete biochemical response

### Biochemical response

**Fig. 1:**
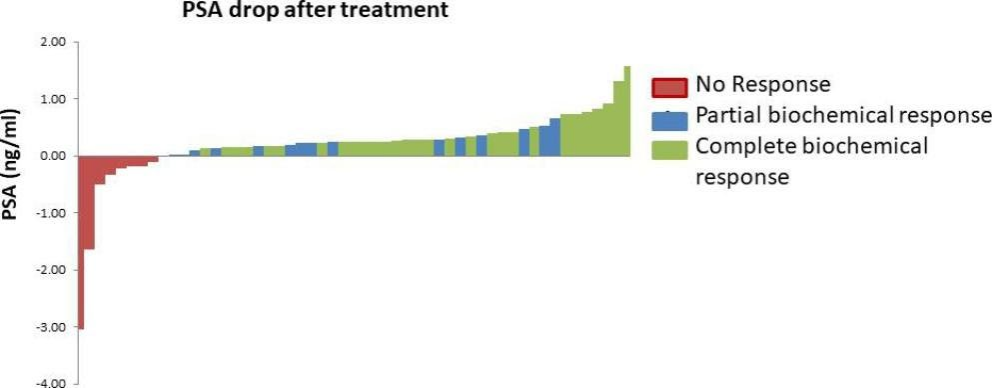
Waterfall diagram summarising PSA drop after treatment *Note: Negative values indicate a PSA progression after treatment.*

Biochemical response data at 3 months after treatment were available for 53 patients. Of these men, 33 (62.3%) had a PBR, out of whom CBR was detected in 29 (54.7%) (Table 2). Median PSA decline was 0.28 ng/ml (IQR 0.19–0.45 ng/ml) (Fig. 1).

Five patients had biochemical progression and underwent a second PSMA re-staging and were found to have distant metastases. Of these men, one had widespread metastatic disease and underwent ADT, three had oligometastatic disease and underwent ADT (n = 2) or a a course of SBRT (n = 1). One patient had negative PSMA imaging result and underwent observation only. Stable disease was reported in 15 men (28.3%).

### Safety

Two patients experienced G2 Genitourinary toxicity. Of these men, one had been treated with prostate bed SRT, and the other underwent SBRT to pelvic nodal disease. Both experienced urinary urgency. There were no G2 or higher Gastrointestinal toxicity.

## Discussion

Overall, the preliminary results from the first cohort of patients enrolled in the PSICHE trial showed promising biochemical outcomes. Of note, only patients with widespread metastatic disease underwent ADT, confirming that a tailored approach based on PSMA findings may maximise benefit of treatment in the salvage setting after RP. Data from EMPIRE-1 trial already showed that clinical outcomes after salvage treatment are significantly improved by the use of NGI [11], but treatment available in that study were limited to prostate bed SRT with or without whole pelvis radiotherapy (for negative PSMA or pelvic positive findings) or RT avoidance for extrapelvic disease. Conversely, other trials tested metastasis directed therapy for oligometastatic disease in a more advanced disease setting, with enrollment exclusive in patients already treated with postoperative salvage or adjuvant prostate bed RT [12]. We believe that use of NGI has a role in earlier scenarios, with the goal to avoid unnecessary overtreatment (e.g. prostate bed radiotherapy when subclinical disease could be detected with NGI). Limitation to the current analysis are related to the early biochemical outcomes reported and the currently limited sample size. Robust data are needed and will be collected with further follow up of the complete cohort.

## Conclusion

The PSICHE trial was designed as a platform to collect prospective data about a treatment strategy encompassing conventional prostate bed SRT for patients with negative NGI, MDT for oligometastatic disease and modern ADT for widespread metastases. Considering the emerging role of NGI, and the lack of standardized approach in current clinical practice, real world clinicalevidence is eagerly awaited to best implement modern RT techniques in post RP salvage setting.

## Electronic supplementary material

Below is the link to the electronic supplementary material.

Supplementary Material 1

Supplementary Material 2

## Abbreviations

SBRT: Stereotactic body radiotherapy
BR: Biochemical relapse
SRT: Salvage radiotherapy
NGI: Next Generation Imaging
PSMA: Prostate Specific Membrane
ADT: Androgen deprivation therapy
AIRO: Italian Association of Radiotherapy and Clinical oncology
CTV: Clinical Target Volume
EORTC: European Organisation for Research and Treatment of Cancer
PTV: Planning Target Volume
OAR: Organs at risk
GTV: Gross target volume
CBR: Complete Biochemical response
PBR: Partial Biochemical Response

## References

[1] Thompson IM, Valicenti RK, Albertsen P, Davis BJ, Goldenberg SL, Hahn C, Klein E, Michalski J, Roach M, Sartor O, Wolf JS Jr, Faraday MM (2013 Aug) Adjuvant and salvage radiotherapy after prostatectomy: AUA/ASTRO Guideline. J Urol 190(2):441–449 10.1016/j.juro.2013.05.032 23707439

[2] Hofman MS, Lawrentschuk N, Francis RJ, Tang C, Vela I, Thomas P, Rutherford N, Martin JM, Frydenberg M, Shakher R, Wong LM, Taubman K, Ting Lee S, Hsiao E, Roach P, Nottage M, Kirkwood I, Hayne D, Link E, Marusic P, Matera A, Herschtal A, Iravani A, Hicks RJ, Williams S, Murphy DG (2020) ; proPSMA Study Group Collaborators. Prostate-specific membrane antigen PET-CT in patients with high-risk prostate cancer before curative-intent surgery or radiotherapy (proPSMA): a prospective, randomised, multicentre study. Lancet. Apr 11;395(10231):1208–1216 10.1016/S0140-6736(20)30314-7 32209449

[3] van den Cornford P, Van den Briers E, Cumberbatch MG, De Santis M, Fanti S, Fossati N, Gandaglia G, Gillessen S, Grivas N, Grummet J, Henry AM, der Kwast THV, Lam TB, Lardas M, Liew M, Mason MD, Moris L, Oprea-Lager DE, der Poel HGV, Rouvière O, Schoots IG, Tilki D, Wiegel T, Willemse PM, Mottet N (2021 Feb) EAU-EANM-ESTRO-ESUR-SIOG guidelines on prostate Cancer. Part II-2020 update: treatment of relapsing and metastatic prostate Cancer. Eur Urol 79(2):263–282 10.1016/j.eururo.2020.09.046 33039206

[4] Van der Deek MP, Sutera P, Deek RA, Fonteyne V, Mendes AA, Decaestecker K, Kiess AP, Lumen N, Phillips R, De Bruycker A, Mishra M, Rana Z, Molitoris J, Lambert B, Delrue L, Wang H, Lowe K, Verbeke S, Van Dorpe J, Bultijnck R, Villeirs G, De Man K, Ameye F, Song DY, DeWeese T, Paller CJ, Feng FY, Wyatt A, Pienta KJ, Diehn M, Bentzen SM, Joniau S, Vanhaverbeke F, De Meerleer G, Antonarakis ES, Lotan TL, Berlin A, Siva S, Ost P, Tran PT (2022 Oct) Long-term outcomes and genetic predictors of response to Metastasis-Directed Therapy Versus Observation in oligometastatic prostate Cancer: analysis of STOMP and ORIOLE trials. J Clin Oncol 10(29):3377–3382 10.1200/JCO.22.00644 PMC10166371 36001857

[5] Francolini G, Di Cataldo V, Detti B, Allegra A, Burchini L, Ciccone LP, Cerbai C, Mattioli C, Frosini G, Guerrieri B, Aquilano M, Salvestrini V, Chiti A, Sollini M, Simontacchi G, Desideri I, Livi L (2022). PO-1363 detection rate of ^68^Ga-PSMA PET/CT from a prospective study: PSICHE trial (NCT05022914). Radiother Oncol.

[6] Rowe SP, Pienta KJ, Pomper MG, Gorin MA, PSMA-RADS, Version (2018 Apr) 1.0: a step towards standardizing the interpretation and reporting of PSMA-targeted PET imaging studies. Eur Urol 73(4):485–487 10.1016/j.eururo.2017.10.027 PMC6859641 29132714

[7] D’Angelillo RM, Francolini G, Ingrosso G, Ravo V, Triggiani L, Magli A, Mazzeo E, Arcangeli S, Alongi F, Jereczek-Fossa BA, Pergolizzi S, Pappagallo GL, Magrini SM (2019 Jun) Consensus statements on ablative radiotherapy for oligometastatic prostate cancer: a position paper of Italian Association of Radiotherapy and Clinical Oncology (AIRO). Crit Rev Oncol Hematol 138:24–28 10.1016/j.critrevonc.2019.03.014 31092381

[8] Poortmans P, Bossi A, Vandeputte K, Bosset M, Miralbell R, Maingon P, Boehmer D, Budiharto T, van den Symon Z, Scrase C, Van Poppel H, Bolla M, EORTC Radiation Oncology Group (2007) Aug;84(2):121-7 ;. Guidelines for target volume definition in post-operative radiotherapy for prostate cancer, on behalf of the EORTC Radiation Oncology Group. Radiother Oncol. 10.1016/j.radonc.2007.07.017 17706307

[9] Marks LB, Yorke ED, Jackson A, Ten Haken RK, Constine LS, Eisbruch A, Bentzen SM, Nam J, Deasy JO Use of normal tissue complication probability models in the clinic. Int J Radiat Oncol Biol Phys. 2010 Mar 1;76(3 Suppl):S10-9. doi: 10.1016/j.ijrobp.2009.07.1754 10.1016/j.ijrobp.2009.07.1754 PMC4041542 20171502

[10] https://www.eortc.be/services/doc/ctc/ctcae_4.03_2010-06-14_quickreference_5x7.pdf

[11] Jani AB, Schreibmann E, Goyal S, Halkar R, Hershatter B, Rossi PJ, Shelton JW, Patel PR, Xu KM, Goodman M, Master VA, Joshi SS, Kucuk O, Carthon BC, Bilen MA, Abiodun-Ojo OA, Akintayo AA, Dhere VR, Schuster DM (2021 May) 18F-fluciclovine-PET/CT imaging versus conventional imaging alone to guide postprostatectomy salvage radiotherapy for prostate cancer (EMPIRE-1): a single centre, open-label, phase 2/3 randomised controlled trial. Lancet 22(10288):1895–1904 10.1016/S0140-6736(21)00581-X PMC8279109 33971152

[12] Hölscher T, Baumann M, Kotzerke J, Zöphel K, Paulsen F, Müller AC, Zips D, Koi L, Thomas C, Löck S, Krause M, Wirth M, Lohaus F (2022 Feb) Toxicity and efficacy of local ablative, image-guided Radiotherapy in Gallium-68 prostate-specific membrane Antigen targeted Positron Emission Tomography-staged, castration-sensitive oligometastatic prostate Cancer: the OLI-P phase 2 clinical trial. Eur Urol Oncol 5(1):44–51 10.1016/j.euo.2021.10.002 34785189

