# Supplementary figures and images for: Early biochemical outcomes following PSMA guided approach for bIoCHEmical relapse after prostatectomy-PSICHE trial (NCT05022914): preliminary results
Source: Clin Exp Metastasis. 2023 Apr 3;40(2):197–201. doi: 10.1007/s10585-023-10204-y (PMC10113311; doi:10.1007/s10585-023-10204-y)

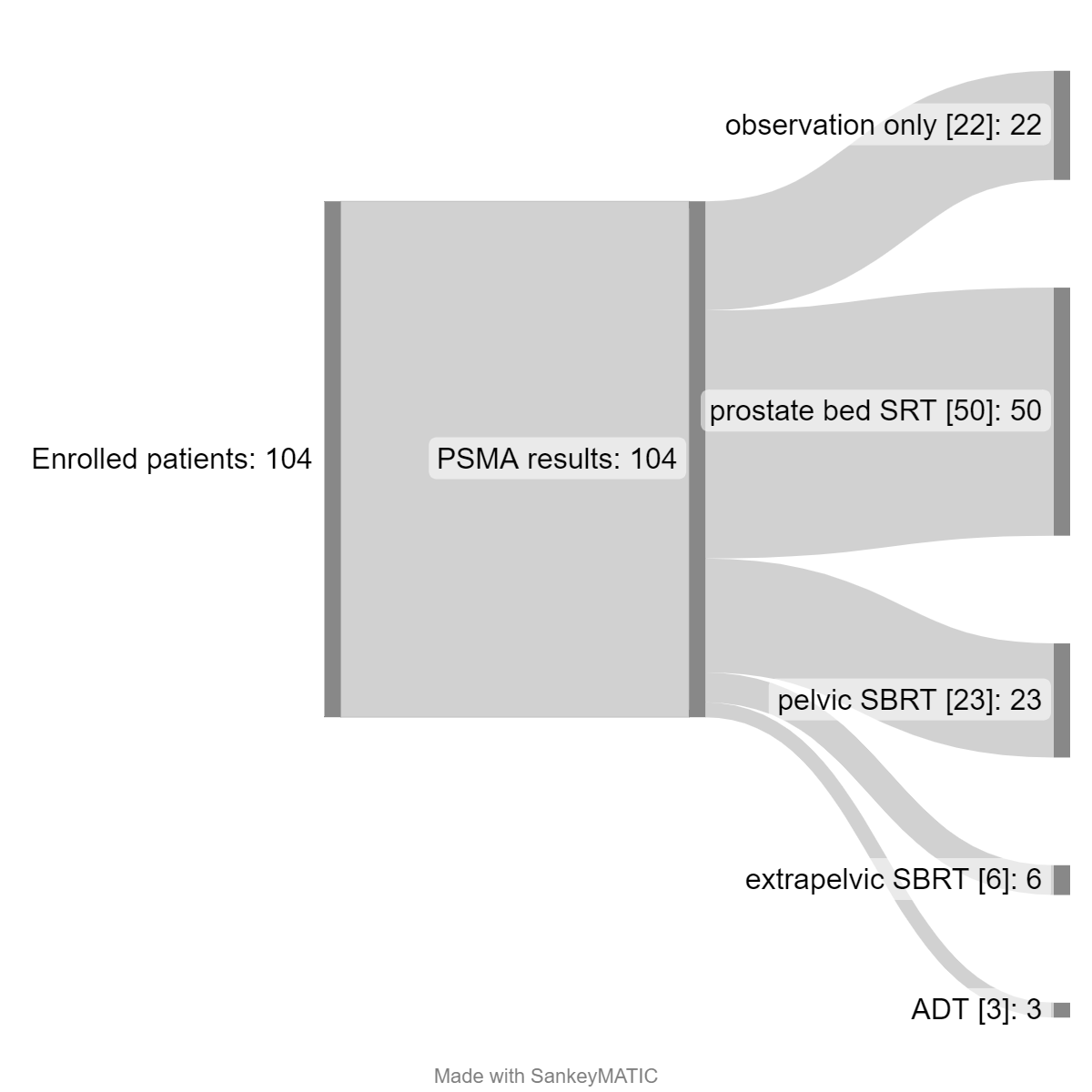

Supplement: Supplementary file 1 — Supplementary Material 1 [file 10585_2023_10204_MOESM1_ESM.png]
