# Supplementary material for: Early biochemical outcomes following PSMA guided approach for bIoCHEmical relapse after prostatectomy-PSICHE trial (NCT05022914): preliminary results
Source: Clin Exp Metastasis. 2023 Apr 3;40(2):197–201. doi: 10.1007/s10585-023-10204-y (PMC10113311; doi:10.1007/s10585-023-10204-y)
Supplement: Supplementary file 2 — Supplementary Material 2 [file 10585_2023_10204_MOESM2_ESM.doc]

**PSMA guided approach for bIoCHEmical relapse after prostatectomy-A prospective observational study-PSICHE**

**Protocol Identifying Number:**

**Protocol Version Number: 1**

**ΕudraCT Number:**

**Sponsor:**

**Dated:**

The investigators declare to be responsible for ensuring that this observational study is performed in accordance with the protocol, concurrent ICH guidelines on Good Clinical Practice (GCP) and applicable regulatory and country­ specific requirements.

In Italy the clinical trials are regulated by regulated by a decree​, entered into force in January 2004 (Decreto legislativo 24 giugno 2003, n.211).This decree transposes the concerning the implementation of in the conduct of clinical trials on medicinal products for human use.

Good Clinical Practice is an international ethical and scientific quality standard for designing, conducting, recording, and reporting studies that involve the participation of human subjects. Compliance with this standard provides public assurance that the rights, safety, and well being of study subjects are protected, consistent with the principles that originated in the Declaration of Helsinki, and that the clinical study data are credible.

Signed:

___________________ ­­­­­__________________

Dott….. Data

___________________ ­­­­­__________________

Dott….. Data

___________________ ­­­­­__________________

Dott….. Data

___________________ ­­­­­__________________

Dott….. Data

**PSMA guided approach for bIoCHEmical relapse after prostatectomy-A prospective observational study-PSICHE**

**1. INTRODUCTION**

**1.1. Background and overall rationale for the study.**

Up to date, salvage prostate bed radiotherapy (SRT) represents the cornerstone therapeutic approach in the setting of early biochemical relapse (BR) after radical prostatectomy (RP) (defined as a detectable or rising PSA value after surgery >0.2 ng/ml) [Thompson 2013///Pisansky 2019///Tendulkar 2016]. However, recent evidence from literature induced European Association of Urology to allow use of 68Ga-PSMA-11 PET/CT to restage all prostate cancer (pCa) patients affected by BR after radical therapy [Cornford 2017], based on its higher accuracy if compared with other metabolic (i.e choline or fluciclofine) imaging [Afshar-Oromieh 2017///Calais 2019]. This prompted significant changes in current management of patients in this setting, leading to the development of imaging-guided approaches [Ost 2015], aimed to postpone palliative androgen deprivation therapy (ADT) [Ost 2018]. Similar approaches already showed to affect clinical management after restaging, but prospective assessment of PSMA-guided therapy to control the disease in the recurrent setting is lacking [Deandreis 2020]. In this setting, different approaches are already available in routine clinical practice (e.g prostate bed radiotherapy, Stereotactic radiotherapy on positive nodal disease or metastatic lesions, androgen deprivation therapy). Prospective evaluation of a standardized approach for these patients could provide interesting insights about the impact of 68Ga-PSMA-11 PET/CT on following clinical outcomes. With this purpose, we designed a prospective, observational study aimed to explore the prognostic factors related to progression free survival after 68Ga-PSMA-11 PET/CT restaging and subsequent treatment. To explore the correlation between PSMA PET/CT findings and predictive biomarkers, aiming to detect a subgroup of patients in whom PSMA PET/CT may have different detection rate, we included a secondary endpoint to evaluate the expression of a specific micro-RNA (miRNA) panel correlated to PSMA gene expression [Erdmann 2014].

**2. OVERVIEW OF STUDY DESIGN**

**2.1. Hypothesis:**

This observational study was designed to evaluate PFS after PSMA-PET/CT based salvage approach for patients affected by BR after RP.

- 1. **Study design**

This is a prospective observational multicenter study including patients treated with upfront RP +/- postoperative prostate bed radiotherapy, with histological result of Prostate adenocarcinoma, affected by BR (defined as PSA > 0.2 ng/ml) with a PSA at recurrence < 1 ng/ml. Patients will be staged with centralized ^68^Ga-PSMA PET/CT and treated with a pre-defined approach based on ^68^Ga-PSMA PET/CT findings, according to routine clinical practice:

| Negative ^68^Ga-PSMA PET/CT or positive findings within prostate bed | Prostate bed RT*, **, *** |
| --- | --- |
| ^68^Ga-PSMA PET/CT detecting pelvic nodal recurrence (defined as nodal disease < 2 cm under aortic bifurcation) amenable with stereotactic body radiation therapy (SBRT) on all sites of disease | SBRT to positive nodal disease |
| Abdominal nodal or bone oligometastatic disease (defined as < 3 non visceral lesions, according to Association of Radiotherapy and Clinical Oncology (AIRO) definition [D’angelillo 2019]) amenable with stereotactic body radiation therapy (SBRT) on all sites of disease | SBRT on all sites of disease |
| Abdominal nodal or bone metastatic disease (> 3 lesions or non-amenable with SBRT) and/or visceral disease. | ADT+/-other systemic therapies available for metastatic hormone sensitive pCa at physician discretion |

**For patients who did not previously received prostate bed RT, dose intensification (e.g 72-74 Gy in 36-37 fractions or equivalent dose/fractionation schedules) will be provided* *in case of positive PSMA/PET-CT findings inside prostate bed. In this case, Stereotactic Salvage RT (e.g. 35 Gy in 5 fractions on macroscopic recurrence) is allowed within site-specific study protocols.*

*** For patients who already received prostate bed RT in postoperative setting with negative findings within prostate bed, observation and PSMA-PET/CT re-staging at further PSA progression (defined as > 25% PSA increase if compared to PSA at recurrence) will be performed*

**** For patients who already received prostate bed RT in postoperative setting with positive findings within prostate bed, both observation and PSMA-PET/CT re-staging at further PSA progression or prostate bed retreatment (e.g SBRT for a total dose of 30 Gy in 5 fractions) will be allowed according to clinician choice*

Planned size of the overall study population is 180 patients. Data about demographics, PSA, comorbidities and current drug therapies up to 45 days prior to enrollment will be collected at baseline. The study will consist in the observation of outcomes after ^68^Ga-PSMA PET/CT staging and following postoperative tailored management. Total planned duration of the study is 84 months, consisting in 36 months enrollment period and a later phase of 48 months in which patients will continue to be submitted to periodic checks every 3 months.

**2.3. Study design rationale**

Standard approach for BR after RP is SRT [Thompson 2013]. However, emerging imaging modalities, such as ^68^Ga-PSMA PET/CT, allowed to effectively estimate location and actual burden of disease, and are currently used in clinical practice. A tailored approach based on ^68^Ga-PSMA PET/CT findings allows to increase clinical outcomes and reduce unnecessary toxicity (e.g prostate bed RT in patients with sublinical metastatic disease, undetectable with conventional staging). However, prospective data about this issue could help to accurately assess prognostic factors associated to clinical outcomes after a tailored postoperative approach.

**3. STUDY OBJECTIVES**

**3.1. Primary objectives**

Primary objective of the study will be to explore prognostic factors correlated with clinical outcomes in the enrolled cohort.

**3.2. Secondary objectives**

-To explore prognostic factors correlated with Overall survival, defined as time between surgery and death

-To record 2 years Health related quality of life

-To record the proportion of management changes induced by positive PSMA PET/CT results

-To assess the association between PSMA PET/CT detection rate and baseline detection of a specific panel of miRNA (miR-186, miR-26a, miR-374a, miR-410, miR-660)

**3.3 Study endpoints**

*Primary endpoint*

Primary objective of the study will be to explore prognostic factors correlated with rate of 2 years PFS. PFS is defined as time from end of salvage treatment performed at first relapse to documented biochemical progression or radiological progression (or both), death from any cause or censoring at date of last follow-up. Biochemical progression is defined as the date in which a PSA increase above 0.2 ng/ml for patients with a PSA nadir < 0.2 ng/ml or 2 consecutive PSA increases >25% if compared to nadir in patients with a PSA nadir > 0.2 ng/ml is detected. Radiological progression will be defined as the occurrence of any new lesion detectable with PSMA PET/CT and/or any other molecular/radiological exam, which will be performed in case of biochemical or clinical progression

*Secondary endpoints*

- Explore prognostic factors correlated with Overall survival (defined as time between end of tailored treatment after ^68^Ga-PSMA PET/CT and death)

- Explore prognostic factors correlated with Cause specific survival, defined as time between end of tailored treatment and death for prostate Cancer

- Explore prognostic factors correlated with Radiological PFS, defined as the occurrence of any new lesion detectable with PSMA

PET/CT and/or any other molecular/radiological exam, which will be performed in case of biochemical or clinical progression

-Record rate of patients with impaired quality of life measured by EORTC QLQ-C30 and EORTC QLQ-PR25 at 2 years (defined as decrease by 10 points in global health scale and functional scales or increase by 10 points in symptoms scales compared with baseline, without later improvement superior by 10 points)

-To assess the proportion of management changes induced by positive PSMA PET/CT results

-To assess the association between PSMA PET/CT detection rate and baseline detection of a specific panel of miRNA (miR-186, miR-26a, miR-374a, miR-410, miR-660)

**4. STUDY POPULATION**

**4.1. Inclusion Criteria**

Each potential subject must satisfy all of the following criteria to be enrolled in the study.

- Signed informed consent
- Age >18
- Patient suitable for 68Ga-PSMA PET/CT re-staging according to clinical practice (Previous radical prostatectomy with histological result of Prostate adenocarcinoma +/-postoperative prostate bed radiotherapy (adjuvant or salvage setting), with a biochemical relapse defined as a PSA > 0.2 and <1

1. **Exclusion Criteria**

- ADT administration within 6 months from studyenrollment
- Persistent elevation of PSA after RP measured within 16 weeks from surgery (> 0.1 ng/ml)

**5. STUDY PROCEDURES**

Patients will be observed after management in order to record clinical outcomes and explore prognostic factors in this cohort. According to clinical practice, the following guideline will be adopted for prostate bed RT and SBRT on all sites of disease (pelvic nodal or oligometastatic)

*1: Prostate bed RT*

Postoperative RT to prostate bed will be administered in case of negative PET PSMA findings or positive findings within prostate bed. Clinical Target Volume (CTV) will be defined according to EORTC guidelines [Poortmans 2007], a CTV to Planning Target Volume (PTV) margin of 5-8 mm will be added according to clinical practice and Image guidance protocols adopted. Both 3D conformal or Intensity modulated RT /Volumetric Modulated Arc Therapy (3D CRT or IMRT/VMAT) are allowed, provided that at least 66 Gy in 33 fractions, or equivalent dose/fractionation schedules, are delivered. Dose intensification (e.g 72-74 Gy in 36-37 fractions or equivalent dose/fractionation schedules) will be provided in case of positive PSMA/PET-CT findings inside prostate bed. At least 95% of PTV should be covered by 95% prescription isodose. Less than 10% of the PTV should be higher than the 105% prescription isodose. Dose constraints to Organs at risk (OAR) should respect QUantitative Analysis of Normal Tissue Effects in the Clinic (QUANTEC) report [Marks 2010]. In case of positive findings within prostate bed in patients who did not received postoperative prostate bed RT, Stereotactic Salvage RT (e.g. 35 Gy in 5 fractions on macroscopic recurrence) is allowed within site-specific study protocols.

*2: SBRT on all sites of disease (pelvic nodal or oligometastatic)*

The GTV will be any demonstrable abdominal nodal or bone metastatic disease detected by PSMA PET/CT. Morphological, topographical and metabolic informations should be integrated to delineate the target volumes. Rigid or deformable co-registration is allowed. No CTV is allowed for SBRT treatments. A GTV to PTV margin of 3-5 mm will be added according to clinical practice and image guidance protocols adopted.

The following dose/fractionation schedules could be adopted to perform SBRT:

| Total dose (Gy) | Dose per fraction (Gy) | Number of fractions |
| --- | --- | --- |
| 24 | 8 | 3 |
| 27 | 9 | 3 |
| 30 | 10 | 3 |
| 36 | 12 | 3 |
| 30 | 6 | 5 |
| 35 | 7 | 5 |
| 40 | 8 | 5 |
| 45 | 9 | 5 |
| 36 | 6 | 6 |
| 45 | 7.5 | 6 |

Dose constraints to Organs at risk (OAR) should respect report of AAPM Task group 101 [Benedict 2010]. A Simultaneous Integrated Protection (SIP) protocol could be adopted to preserve critical OAR.

**6. STUDY EVALUATIONS**

**6.1 Pre­Treatment Evaluation**

Baseline evaluations include:

- - demographics data;
  - active co­morbidities and current medical treatments;
  - Pathological staging, margin status, PSA value and Gleason Score at the time of prostate cancer diagnosis;
  - previous treatments for prostate cancer;
  - Baseline PSA at BR
  - Imaging procedures:
- Staging with PSMA PET/CT
  - assessment of Performance Status with the Eastern Cooperative Oncology Group (ECOG) Score;
  - Baseline Quality of life assessment measured by EORTC QLQ-C30 and EORTC QLQ-PR25 (baseline evaluation should be performed before radiotherapy beginning);
  - Blood samples to detect baseline expression of a specific panel of miRNA (miR-186, miR-26a, miR-374a, miR-410, miR-660)
  1. **Evaluation During Treatment**

Patients will be evaluated as follows:

- After initial PSMA PET/CT, treatment modalitywill be recorded to assess the proportion of management changes induced by positive PSMA PET/CT results
- every 3 months from therapy start with ECOG Score EORTC QLQ-C30 and EORTC QLQ-PR25
- PSA value every 3 months from therapy start
- PSMA PET/CT will be repeated in case of biochemical progression, defined as a PSA increase above 0.2 ng/ml for patients with a PSA nadir < 0.2 ng/ml or 2 consecutive PSA increases >25% if compared to nadir in patients with a PSA nadir > 0.2 ng/ml is detected.

**6.3 Statistical Analysis**

Considering the observational nature of the present protocol, a strict criterion for a target accrual would not be needed. However considering a reported rate for 2 year progression free survival after salvage radiotherapy alone of 80% [Carrie 2019], a sample size of 172 patients would be needed to estimate with a 6% precision margin and a 95% confidence interval. Considering a 5% drop out rate, 180 patient are the target accrual fixed for this study. In case this target could not be achieved, smaller sample size would lead to 95% confidence intervals ranging 73-86% (for N=150), 72-87% (for N=120), and 70-88% (for N=90), i.e. with a precision margin still within 10% (always assuming a 2-y PFS equal to 80%). Furthermore, considering that PSMA PET/CT has been shown to change therapeutic management in 34% of cases [DeAndreis 2020], 90 patients would be sufficient to assess with a 11% precision margins the rate of patients in whom re-staging allowed to tailor management. Considering the explorative nature of the translational objectives of the study, a target sample size will not be needed.

**7. SAFETY**

**Adverse Events (AE)**

Every clinical harmful event occurring in a patient involved in a study, related or not to the treatment object of the study. Every subject reporting an adverse event will be examined as soon as possible by a study investigator. Investigator will do all the necessary to ensure safety and wellness of subject, until the complete resolution or stabilization of adverse event.

**Serious Adverse Events (SAE)**

A serious adverse event is every event satisfying at least one of these criteria:

It results in death

It is life threatening

It requires of hospitalisation or prolonged recovery resulting in invalidity or severe/prolonged incapability

It determines a congenital anomaly or birth defect

**Adverse Device effect (ADE)**

An Adverse Device Effect (ADE) is every adverse event related to the use of a medical experimental device. ADEs regard also:

- every adverse event results from incompleteness or inaccuracy of instructions or every malfunction of device
- every event resulting from an error of use or intentionally improper use

ADEs are severe **(SADE)**, when resulting in classic serious a dverseevents.

ADEs are unexpected and severe (USADE) when they are unpredictable based on known risk profile of device in study.

**Management of risks related to study intervention**

**Urethral stricture:** It is one of the most common side effects of radiotherapy. Strictures are mostly located at the bulbomembranous part of the urethra. Diagnostic work-up should include basic urologic work-up, ultrasound, uroflowmetric assessment, urethroscopy, retrograde urethrogram and voiding cystourethrography. Endoscopic management such as dilatation and internal urethrotomy has been proposed in short strictures. However these therapies have a high risk for recurrence. The success rate of urethroplasty is higher.

**Cystitis:** is a complication of radiation therapy to pelvic tumors. Manifestations of radiation cystitis can range from minor, temporary, irritative voiding symptoms and painless, microscopic hematuria to more severe complications, such as gross hematuria; contracted, nonfunctional bladder; persistent incontinence; fistula formation; necrosis.

**Proctitis, Rectal bleeding, Bowel Symptoms:** Experiencing symptoms of diarrhea, urgency, incontinence, and rectal bleeding can be a significant source of stress. Those individuals with a history of prior radiation therapy exposure are at risk to develop this kind of gastrointestinal discomfort.

**Urinary incontinence:** Incontinence is the unwanted leakage of urine. This symptom is often reported after prostatectomy, however, urinary incontinence may be significantly affected after prostate radiotherapy

**Erectile disfunction:** Sexual function may be significantly affected after radical prostatectomy, however, erectile disfunction may be enhanced after prostate radiotherapy

**8.ETHICAL ASPECTS**

**8.1 Ethical aspects and Confidentiality**

Investigators ensure full study conformity according to international normative and its national application, respecting principles of Helsinki Declaration in order to assure maximum protection of involved subjects. Principal Investigator ensure clinical study conformity to protocol and Good Clinical Practice (GCP). Study promoter guarantees the protection of clinical and not-clinical sensitive personal data according to national normative [D.Lvo. 196/2003].

**8.2 Informed consent**

Investigators must obtain informed consent after adequate discussion about purposes, methods, expected benefits and predictable risks of the study. Investigators must elucidate subjects about the fact that voluntary exclusion or interruption of participation will not cause any prejudice or damage.

**8.3 Ethical Committee and Competent Authorities**

Promoter will provide to reference Ethical Committeethe study protocol and every other document provided to the patient (Informative Note and Informed Consent Format). Ethical Committee and competent authority approval has to be obtained before the beginning of every study-related procedure, and it has to be documented through official communication to investigators. In variations of protocol during the clinical study will be needed, promoter will present adequate demand for amendment to protocol to reference Ethical Committee; its approval will follow procedures defined by Ethical Committee regulations.

**8.4 Data ownership**

Data ownership belongs to Promoter, according to independence of the study defined by D.M. 17-12-2004 (D.M. 17-12-2004, Art. 1, comma 2, c letter)

**8.5 Final report and publication of results**

According to ICH-GCP, principal investigator ensure that the study report, the publication of all collected data as described in the protocol will be responsibly and coherently reported. Publication will occur regardless the obtained results. Data diffusion will happen exclusively after their statistical elaboration, through scientific publications and/or congress talks, seminaries, participation to multicentric studies, and anonymously. Dr Lorenzo Livi is responsible of entire research and data use.

**8.6 Independence of the study**

The Study presents all requirements according to D.M. 17-12-2004 (Art.1, Comma 1 e 2), to be defined as “clinical investigation finalized to the improvement of clinical practice as integral part of health care, not for industrial purposes”.

**12.REFERENCES**

1. Mottet N, Bellmunt J, Bolla M, Briers E, Cumberbatch MG, De Santis M, Fossati N, Gross T, Henry AM, Joniau S, Lam TB, Mason MD, Matveev VB, Moldovan PC, van den Bergh RC, Van den Broeck T, van der Poel HG, van der Kwast TH, Rouvière O, Schoots IG, Wiegel T, Cornford P. [EAU-ESTRO-SIOG Guidelines on Prostate Cancer. Part 1: Screening, Diagnosis, and Local Treatment with Curative Intent.](https://www.ncbi.nlm.nih.gov/pubmed/27568654) Eur Urol. 2017 Apr;71(4):618-629.
2. Thompson IM, Valicenti RK, Albertsen P, Davis BJ, Goldenberg SL, Hahn C, Klein E, Michalski J, Roach M, Sartor O, Wolf JS Jr, Faraday MM. Adjuvant and salvage radiotherapy after prostatectomy: AUA/ASTRO Guideline. J Urol. 2013 Aug;190(2):441-9.
3. Pisansky TM, Thompson IM, Valicenti RK, D'Amico AV, Selvarajah S. Adjuvant and Salvage Radiotherapy after Prostatectomy: ASTRO/AUA Guideline Amendment 2018-2019. J Urol. 2019 May.
4. Tendulkar RD, Agrawal S, Gao T, Efstathiou JA, Pisansky TM, Michalski JM, Koontz BF, Hamstra DA, Feng FY, Liauw SL, Abramowitz MC, Pollack A, Anscher MS, Moghanaki D, Den RB, Stephans KL, Zietman AL, Lee WR, Kattan MW, Stephenson AJ. Contemporary Update of a Multi-Institutional Predictive Nomogram for Salvage Radiotherapy After Radical Prostatectomy. J Clin Oncol. 2016 Oct 20;34(30):3648-3654.
5. Cornford P, Bellmunt J, Bolla M, Briers E, De Santis M, Gross T, et al. EAU-ESTRO-SIOG guidelines on prostate cancer. Part II: treatment of relapsing, metastatic, and castration-resistant prostate cancer. Eur Urol. 2017;71(4):630–42.
6. Afshar-Oromieh A, Holland-Letz T, Giesel FL, et al. Diagnostic performance of 68Ga-PSMA-11 (HBED-CC) PET/CT in patients with recurrent prostate cancer: evaluation in 1007 patients [published correction appears in Eur J Nucl Med Mol Imaging. 2017 Sep;44(10 ):1781]. Eur J Nucl Med Mol Imaging. 2017;44(8):1258–68.
7. Calais J, Ceci F, Eiber M, Hope TA, Hofman MS, Rischpler C, et al. (18)F-fluciclovine PET-CT and (68)Ga-PSMA-11 PET-CT in patients with early biochemical recurrence after prostatectomy: a prospective, single-centre, single-arm, comparative imaging trial. Lancet Oncol. 2019.
8. Ost P, Bossi A, Decaestecker K, De Meerleer G, Giannarini G, Karnes RJ, et al. Metastasis-directed therapy of regional and distant recurrences after curative treatment of prostate cancer: a systematic review of the literature. Eur Urol. 2015;67(5):852–63.
9. Ost P, Reynders D, Decaestecker K, Fonteyne V, Lumen N, De Bruycker A, Lambert B, Delrue L, Bultijnck R, Claeys T, Goetghebeur E, Villeirs G, De Man K, Ameye F, Billiet I, Joniau S, Vanhaverbeke F, De Meerleer G. Surveillance or Metastasis-Directed Therapy for Oligometastatic Prostate Cancer Recurrence: A Prospective, Randomized, Multicenter Phase II Trial. J Clin Oncol. 2018 Feb 10;36(5):446-453.
10. Deandreis D, Guarneri A, Ceci F, Lillaz B, Bartoncini S, Oderda M, Nicolotti DG, Pilati E, Passera R, Zitella A, Bellò M, Parise R, Carlevato R, Ricardi U, Gontero P. (68)Ga-PSMA-11 PET/CT in recurrent hormone-sensitive prostate cancer (HSPC): a prospective single-centre study in patients eligible for salvage therapy. Eur J Nucl Med Mol Imaging. 2020 Apr 20.
11. Erdmann K, Kaulke K, Thomae C, et al. Elevated expression of prostate cancer-associated genes is linked to down-regulation of microRNAs. BMC Cancer. 2014;14:82.
12. D’Angelillo RM, Francolini G, Ingrosso G, Ravo V, Triggiani L, Magli A, Mazzeo E, Arcangeli

S, Alongi F, Jereczek-Fossa BA, Pergolizzi S, Pappagallo GL, Magrini SM. Consensus statements on ablative radiotherapy for oligometastatic prostate cancer: A position paper of Italian Association of Radiotherapy and Clinical Oncology (AIRO). Crit Rev Oncol Hematol. 2019 Jun;138:24-28.

1. Carrie C, Magné N, Burban-Provost P, Sargos P, Latorzeff I, Lagrange JL, Supiot S, Belkacemi Y, Peiffert D, Allouache N, Dubray BM, Servagi-Vernat S, Suchaud JP, Crehange G, Guerif S, Brihoum M, Barbier N, Graff-Cailleaud P, Ruffion A, Dussart S, Ferlay C, Chabaud S. Short-term androgen deprivation therapy combined with radiotherapy as salvage treatment after radical prostatectomy for prostate cancer (GETUG-AFU 16): a 112-month follow-up of a phase 3, randomised trial. Lancet Oncol. 2019 Dec;20(12):1740-1749.
